# Effectiveness of epicardial atrial pacing using a bipolar steroid-eluting endocardial lead with active fixation in an experimental model

**DOI:** 10.1186/1749-8090-8-S1-O56

**Published:** 2013-09-11

**Authors:** SCP Bueno, WT Tamaki, MFA Silva, CM Zambolim, KR Silva, M Martinelli Filho, R Costa, FB Jatene

**Affiliations:** 1 Cardiovascular Surgery Department, Heart Institute (InCor), Hospital das Clínicas da Faculdade de Medicina da Universidade de São Paulo, São Paulo, Brazil

## Background

Although transvenous access for atrioventricular pacemaker implantation is considered the state of the art, clinical and technical situations that may impede transvenous leads implantation have become increasingly common, making it necessary the proposal of new surgical approaches. The aim of this study was to assess the effectiveness of bipolar epicardial atrial pacing using an active fixation bipolar endocardial lead implanted on the atrial surface in an experimental model.

## Methods

A total of 10 large white adult pigs underwent pacemaker implantation under general anesthesia. Atrial pacing and sensing parameters were obtained at the procedure, immediate postoperative period and on the 7th and the 30th postoperative in unipolar and bipolar configurations.

## Results

All procedures were successfully performed. There were no perioperative complications and no early deaths. Atrial pacing and sensing parameters for both unipolar and bipolar modes remained stable throughout the study. We observed a progressive increase in atrial thresholds, ranging from 0.49 ± 0.35 (at implantation) to 1.86 ± 1.31 volts (30th postoperative day), in unipolar mode. Atrial impedance measurements decreased slightly over time, ranging from 486.80 ± 126.35 Ohms (at implantation) to 385.0 ± 80.52 Ohms (30th postoperative day). Atrial sensing measures remained stable from the immediate postoperative period until the end of the study.

## Conclusion

The results of this study demonstrate that a bipolar active fixation endocardial lead implanted epicardially can provide stable conditions of pacing and sensing parameters throughout the postoperative follow-up.

